# Distinct Genetic Diversity of *Oncomelania hupensis*, Intermediate Host of *Schistosoma japonicum* in Mainland China as Revealed by ITS Sequences

**DOI:** 10.1371/journal.pntd.0000611

**Published:** 2010-03-02

**Authors:** Qin Ping Zhao, Ming Sen Jiang, D. Timothy J. Littlewood, Pin Nie

**Affiliations:** 1 Department of Parasitology, School of Basic Medical Science, Wuhan University, Wuhan, Hubei Province, China; 2 State Key Laboratory of Freshwater Ecology and Biotechnology, Institute of Hydrobiology, Chinese Academy of Sciences, Wuhan, Hubei Province, China; 3 Department of Zoology, The Natural History Museum, Cromwell Road, London, United Kingdom; University of Queensland, Australia

## Abstract

**Background:**

*Oncomelania hupensis* is the unique intermediate host of *Schistosoma japonicum*, which causes schistosomiasis endemic in the Far East, and especially in mainland China. *O. hupensis* largely determines the parasite's geographical range. How *O. hupensis*'s genetic diversity is distributed geographically in mainland China has never been well examined with DNA sequence data.

**Methodology/Principal Findings:**

In this study we investigate the genetic variation among *O. hupensis* from different geographical origins using the combined complete internal transcribed spacer 1 (ITS1) and ITS2 regions of nuclear ribosomal DNA. 165 *O. hupensis* isolates were obtained in 29 localities from 7 provinces across mainland China: lake/marshland and hill regions in Anhui, Hubei, Hunan, Jiangxi and Jiangsu provinces, located along the middle and lower reaches of Yangtze River, and mountainous regions in Sichuan and Yunnan provinces. Phylogenetic and haplotype network analyses showed distinct genetic diversity and no shared haplotypes between populations from lake/marshland regions of the middle and lower reaches of the Yangtze River and populations from mountainous regions of Sichuan and Yunnan provinces. The genetic distance between these two groups is up to 0.81 based on *F*st, and branch time was estimated as 2–6 Ma. As revealed in the phylogenetic tree, snails from Sichuan and Yunnan provinces were also clustered separately. Geographical separation appears to be an important factor accounting for the diversification of the two groups of *O. hupensis* in mainland China, and probably for the separate clades between snails from Sichuan and Yunnan provinces. In lake/marshland and hill regions along the middle and lower reaches of the Yangtze River, three clades were identified in the phylogenetic tree, but without any obvious clustering of snails from different provinces.

**Conclusions:**

*O. hupensis* in mainland China may have considerable genetic diversity, and a more complex population structure than expected. It will be of significant importance to consider the genetic diversity of *O. hupensis* when assessing co-evolutionary interactions with *S. japonicum*.

## Author Summary

The intermediate host of *Schistosoma japonicum* in Asia is the snail *Oncomelania hupensis*, which can be separated phenotypically into ribbed- and smooth-shelled morphotypes. In China, the typical morphotype is ribbed-shelled, with its distribution restricted to mainland China. Smooth-shelled snails with varix are also distributed in China, which are considered to belong to the same subspecies as the ribbed-shelled snails. In this study we investigate the genetic variation among *O. hupensis* from different geographical origins using combined complete ITS1 and ITS2 regions of nuclear ribosomal DNA. Snails including ribbed-shelled and smooth-shelled (but with varix on the shell) from the lake/marshland region of the middle and lower reaches of the Yangtze River, and smooth-shelled snails from mountainous regions of Sichuan and Yunnan provinces, were genetically distinct with no shared haplotypes detected. Furtheremore, the snails from Sichuan and Yunnan provinces were clustered in separate clades in the phylogenetic tree, and three clades were observed for snails from the middle and lower reaches of the Yangtze River. The population diversity of *O. hupensis* in China is thus considered large, and evolutionary relationships in the host-parasite system of *O. hupensis*-*S. japonicum* may be of interest for further research.

## Introduction

The snail *Oncomelania hupensis*, the only intermediate host of *Schistosoma japonicum*, has been found in China, and also in Japan, Philippines and Indonesian island of Sulawesi. Over the past a few decades, the taxonomy of *O. hupensis* has been a dispute due to the variation in morphological characters such as shell sculpture, operculum etc. [1]–[3]. Phenotypically, *O. hupensis* can be separated into ribbed- and smooth- shelled morphotypes. In China, the typical morphotype of *O. hupensis* is ribbed-shelled, and its distribution is restricted to Yangtze River basin. Smooth-shelled snails are also distributed in mainland China, but are considered as the same species and subspecies of *O. hupensis* [1]–[4]. *Oncomelania* snails reported in other Far East countries are smooth-shelled, and have been considered either as subspecies of *O. hupensis* or independent species in this genus [5]–[8].

Based on shell form, biogeographical and allozyme data, Davis et al. [1] distinguished all of the *O. hupensis* in mainland China into three subspecies: *O. hupensis* subsp. *robertsoni*, *O. hupensis* subsp. *tangi* and *O. hupensis* subsp. *hupensis*. *O. hupensis robertsoni* which has a small, smooth shell but with no varix, is found in Sichuan and Yunnan provinces. *O. hupensis tangi*, which has a smooth shell but with thick varix, is found in Fujian province and Guangxi autonomous region, separated geographically from the Yangtze River, and extensive control measures have brought this subspecies to near extinction [9],[10]. However, Zhou et al. [11] separated the *O. hupensis guangxiensis* out from *O. hupensis* subsp. *tangi* based on allozymes and amplified fragment length polymorphism (AFLP) [12],[13] , which was verified recently by Li et al. [14] with internal transcribed spacer (ITS) and 16S fragments. *O. hupensis hupensis* is the most widely distributed subspecies of *Oncomelania* and lives primarily at low altitude but a few populations live in hilly areas in the drainage area of the Yangtze River in mainland China. It has varix, no matter whether the shell is smooth or ribbed, but most populations have ribbed-shell. *O. hupensis hupensis* has the same shell growth allometry as *O. hupensis robertsoni* but has a longer shell on average [1],[2].

The genetic diversity of *O. hupensis* in China has also been a focus over last two decades, and some results have been controversial. Spolsky et al. [15], by using cyt b gene, found considerable genetic diversity in *O. hupensis* in China, and using AFLP, Zhou et al. [12],[13] detected significant positive correlation between genetic and geographical distances for 25 populations of *O. hupensis* collected in China. With allozyme data, Davis et al. [1] showed that one smooth-shelled population from Zhejiang province could be considered genetically identical to a population of *O. hupensis robertsoni* from Sichuan province. Also, using an allozyme approach, Zhou et al. [16] and Qian et al. [17] found that smooth-shelled populations were clustered separately with ribbed-shelled populations in middle and lower reaches of the Yangtze River. With mitochondrial cytochrome oxidase subunit 1 (cox1) gene, Wilke et al. [3] showed that smooth-shelled individuals clustered together with ribbed-shelled ones, all collected in the middle and lower reaches of the Yangtze River, suggesting that all smooth- and ribbed- shelled populations of *Oncomelania* throughout the middle and lower Yangtze River basin belong to the subspecies *O. hupensis hupensis*. With the 16S RNA and ITS sequences respectively, Li et al. [14] recently found four and three branches in the phylogenetic trees, with the four branches representing *O. hupensis robertsoni* from Sichuan and Yunnan provinces, *O. hupensis guangxiensis* from Guangxi Karst region, *O. hupensis hupensis* from the middle and lower reaches of Yangtze River, and those from littoral and hill regions in Fujian province which was recognized as *O. hupensis tangi* [1]. However, the report by Li et al. [14] contained only a small number of specimens. Comprehensive analyses on the genetic diversity of these snails and the relationship between *O. hupensis hupensis* in the middle and lower Yangtze River basin and the smooth-shelled *O. hupensis robertsoni* in areas of upper Yangtze River have not been carried out with more samples collected on a much larger geographical scale.

In this study, the intermediate hosts of *S. japonicum* were collected from 29 localities in 7 provinces, comprising almost all uncontrolled endemic areas of schistosomiasis in mainland China. *O. hupensis hupensis* and *O. hupensis robertsoni* were obtained from localities in the middle and lower reaches of the Yangtze River, and from Sichuan and Yunnan Provinces in the upper Yangtze River, respectively. Highly variable internal transcribed spacer regions (ITS) of nuclear ribosomal DNA were sequenced for individual *O. hupensis* snails in order to examine the genetic diversity of *O. hupensis hupensis* and *O. hupensis robertsoni* in mainland China, and to find out the relationship between their geographical distribution and the genetic variation of these snails in China on the basis of phylogenetic analysis. The evolutionary implication of the intermediate host genetic diversity was then discussed.

## Materials and Methods

### Snail specimens

**Figure 1:**
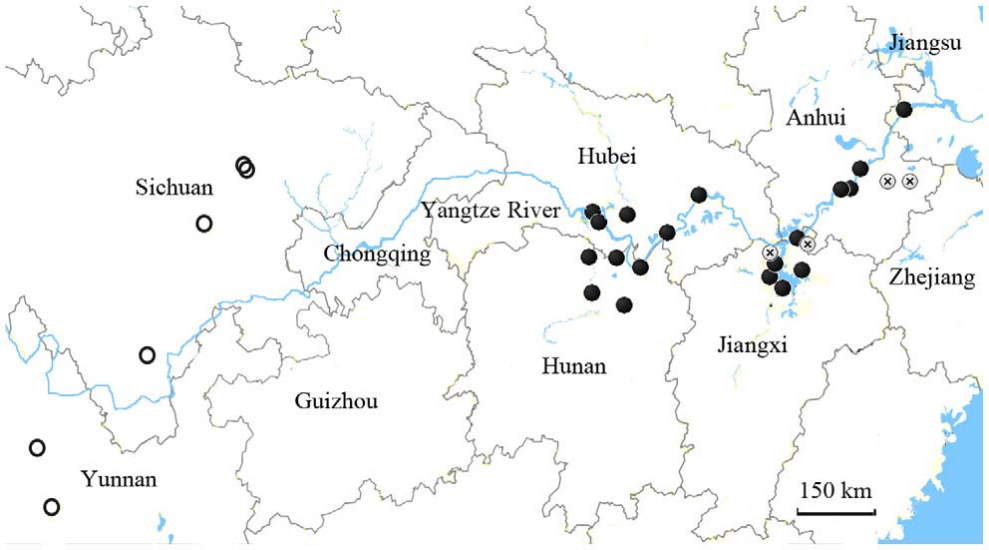
Sample localities indicated in provinces of China. Samples of ribbed-shelled snails are indicated as •, smooth-shelled with varix as ⊗, and smooth-shelled as ◯.

The diagnosis of subspecies of *O. hupensis* followed that of Davis et al. [1]. *O. hupensis hupensis* and *O. hupensis robertsoni* were collected from October 2005 to October 2006 from endemic areas in Anhui, Hubei, Hunan, Jiangxi, Jiangsu, and in Sichuan and Yunnan provinces in mainland China, respectively (Table 1). Geographical information concerning these sample localities is listed in Table 1 and indicated in Fig. 1 using Google Earth with editing in Photoshop. Snails were collected with forceps from the field and brought back to laboratory, where they were cleaned after one month captivity, and then checked microscopically to ensure that schistosome-uninfected snails were selected for the experiment. The head-foot muscle of each snail was dissected individually under a microscope after being washed in 0.3% NaCl solution, and then preserved in 95% ethanol.

**Table 1:** List of population samples in the study, with collection data and haplotype information for *Oncomelania hupensis* ITS1–ITS2 sequences.

Locality*	Province	Latitude, longitude	Altitude (m)	Shell	N	Haplotype
Wuhan (HBwh)	Hubei	30°38′N; 114°20′E	18	Ribbed	5	H42, 43, 54, 63, 64
Jingzhou (HBjz)	Hubei	30°20′N; 112°02′E	31	Ribbed	10	H27, 55–57, 61, 68, 75–77, 80
Qianjiang (HBqj)	Hubei	30°17′N; 112°47′E	20	Ribbed	7	H26, 28, 69, 72, 78
Gong'an (HBga)	Hubei	30°09′N; 112°10′E	42	Ribbed	7	H46, 53, 59, 62, 64, 81
Honghu (HBhh)	Hubei	29°58′N; 113°39′E	13	Ribbed	5	H1, 48, 60, 65, 74
Xiangyin (HBxy)	Hunan	28°41′N; 112°43′E	27	Ribbed	9	H16, 20, 22, 23, 34, 41, 65
Lixian (HNlx)	Hunan	29°32′N; 111°57′E	38	Ribbed	6	H18, 32, 33, 45, 52, 79
Hanshou (HNhs)	Hunan	28°54′N; 112°01′E	31	Ribbed	6	H16, 23, 44, 65, 70, 73
Yueyang (HNyy)	Hunan	29°21′N; 113°04′E	27	Ribbed	5	H16, 17, 65, 70
Huarong (HNhr)	Hunan	29°31′N; 112°33′E	32	Ribbed	5	H65, 66
Xuanzhou (AHxz)	Anhui	30°53′N; 118°54′E	45	Smooth, varix	8	H38, 40
Nanling (AHnl)	Anhui	30°53′N; 118°25′E	122	Smooth, varix	5	H14, 37, 47, 49
Tongling (AHtl)	Anhui	31°06′N; 117°50′E	89	Ribbed	4	H2, 4, 6
Guichi (AHgc)	Anhui	30°45′N; 117°37′E	108	Ribbed	5	H4, 35, 50, 71
Zongyang (AHzy)	Anhui	30°44′N; 117°25′E	5	Ribbed	4	H8, 9, 10, 11
Gongqin (JXgq)	Jiangxi	29°11′N; 115°52′E	8	Ribbed	4	H8, 39, 50
Xingzi (JXxz)	Jiangxi	29°25′N; 115°59′E	45	Ribbed	5	H8, 12, 13, 50,
Jiujiang (JXjj)	Jiangxi	29°37′N; 115°52′E	31	Smooth, varix	5	H4, 5, 7, 15
Pengze (JXpz1)	Jiangxi	29°52′N; 116°28′E	16	Ribbed	4	H24, 58, 67, 73
Pengze (JXpz2)	Jiangxi	29°46′N; 116°41′E	52	Smooth, varix	6	H18, 19
Poyang (JXpy)	Jiangxi	29°18′N; 116°34′E	28	Ribbed	5	H21, 25, 29, 31, 36
Xinjian (JXxj)	Jiangxi	28°59′N; 116°09′E	−19	Ribbed	5	H4, 13, 30, 51
Nanjing (JSnj)	Jiangsu	32°09′N; 118°47′E	−3	Ribbed	5	H3, 4, 6
Jingyang (SCjy)	Sichuan	31°09′N; 104°29′E	592	Smooth	6	H82, 83
Zhongjiang(SCzj)	Sichuan	31°06′N; 104°32′E	675	Smooth	6	H83, 84
Meishan (SCms)	Sichuan	30°07′N; 103°36′E	524	Smooth	5	H85, 86, 87
Xichang (SCxc)	Sichuan	27°49′N; 102°22′E	1748	Smooth	6	H88, 89
Weishan (YNws)	Yunnan	25°06′N; 100°18′E	1888	Smooth	5	H86, 91–94
Eryuan (YNey)	Yunnan	26°09′N; 99°59′E	2105	Smooth	7	H90

*Each locality is designated with a two-letter province code followed by two-letter city or county code. For JXpz1 and JXpz2, the number means different locality from the same county where snail shells have different types.

N, number of snails sampled in this location.

### DNA extraction, PCR amplification, and sequencing of ITS

The total genomic DNA of individual snails was extracted using a standard sodium dodecyl sulfate-proteinase K procedure [18]. Each individual sample was incubated and thawed in 200 µl extraction buffer (50 mM Tris-HCl, 50 mM EDTA, 100 mM NaCl, 1% SDS, 100 µg/ml proteinase K), at 56°C for 2 h with gentle mixing. DNA in solution was extracted using standard phenol/chloroform purification, followed by 3 M sodium acetate (pH 5.2) and ethanol precipitation. Pellets of DNA were washed in 70% ethanol, air-dried, and resuspended in 20 µl TE (pH 8.0). Polymerase chain reaction (PCR) was used to generate a fragment spanning ITS1-5.8S-ITS2 between the forward primer OHITSF (5′- ATTGAACGGTTTAGTGAGGTCC -3′) and the reverse primer OHITSR (5′- CATTCCCAAACAACCCGACTC -3′) based on available GenBank sequences AY207042, AF367667 and U93228. The PCR protocols were 94°C for 3 min followed by 30 cycles of 94°C for 30s, 58°C for 30s, and 72°C for 90 s and then a final elongation step at 72°C for 10 min. The amplified products were purified on a 1.0% agarose gel stained with ethidium bromide, using the DNA gel extraction kit (Omega Bio-Tek). The purified PCR product was then cloned into pMD18-T vector (TAKARA) and sequenced using ABI PRISM BigDye Terminators v3.0 Cycle Sequencing (Applied Biosystems). The DNA sequences were deposited in the GenBank database under accession numbers FJ600745 to FJ600909 inclusive.

### Sequence alignments and analyses

Sequences were aligned using ClustalX v1.83 [19] at default settings followed by manual correction in SEAVIEW [20]. DNAsp version 4.0 [21] was used to define the haplotypes.

Genetic variation within and between two subspecies were estimated by calculating nucleotide diversity (π) and haplotypic diversity (*h*) values in Arlequin3.11 [22] and DNAsp. Selective neutrality was tested with Tajima's *D* [23] and Fu's *F* test [24].

Phylogenetic relationships were conducted on the aligned sequences of combined ITS1-ITS2 rDNA sequences. We performed a wide array of phylogenetic analyses using different methods: neighbor joining (NJ), maximum parsimony (MP), maximum likelihood (ML) and Bayesian inference (BI). NJ and MP were implemented in PAUP^*^ 4.0b10 [25] using heuristic searches and tree bisection-reconnection branch-swapping. Nodal support for the MP phylogenetic tree was estimated through bootstrap analysis using 1000 replicates, and with 10 random sequence additions per each step bootstrap replicates. ML analysis was conducted in PHYML 2.4.4 [26], also with 1000 replicates bootstrap. GTR+I+G was determined as the best-fit model of sequence evolution for each dataset by using the Akaike informative criterion implemented in Modeltest 3.7 [27]. BI was carried out with MrBayes 3.1 [28] under the best-fit substitution model. Analyses were run for 2×10^6^ generations with random starting tree, and four Markov chains (with default heating values) sampled every 100 generations. Posterior probability values were estimated by generating a 50% majority rule consensus tree after the first 2000 trees were discarded as part of a burn-in procedure. All phylogenetic trees were rooted using *Lottia digitalis* as outgroup.

Mismatch distribution of the number of differences between all possible pairs of haplotypes were calculated using DNAsp, and tested against the expected values of a recent population expansion with 1000 bootstrap replicates. Within-species genetic structure was phylogenetically evaluated by constructing unrooted parsimony networks of haplotypes using TCS version 1.21 [29]. Net nucleotide divergence (D_xy_) between two subspecies was calculated with the Tamura-Nei gamma correction model using MEGA 4 [30].

## Results

### Sequence variation and genetic diversity

The complete ITS-5.8S-ITS2 fragments, including portions of the 3′ end of the 18S and 5′ start of the 28S, were sequenced for individual snails. The 3′ part of the 18S, 5′ part of the 28S and 5.8S of all specimens are completely identical. The ITS1 and ITS2 regions ranged from 412 to 441 bp and from 402 to 426 bp, respectively. The alignment of the combined ITS1–ITS2 sequences resulted in a total of 889 characters, including gaps, with 190 variable sites and 71 parsimony informative sites. A total of 93 haplotypes were identified from 165 individuals. 31 haplotypes were found in multiple individuals and 62 haplotypes were represented by single individuals (Table 1). The haplotype and nucleotide diversity for all sequences sampled were 0.974±0.004 and 0.023±0.002, respectively.

For *O. hupensis hupensis*, 80 haplotypes were identified from 130 individuals in 23 localities of five provinces along middle and lower reaches of Yangtze River. The haplotype and nucleotide diversity were 0.960±0.022 and 0.017±0.008, respectively. For *O. hupensis robertsoni*, 13 haplotypes identified from 35 individuals of 6 localities in Sichuan and Yunnan provinces. The haplotype and nucleotide diversity were 0.916±0.023 and 0.028±0.014, respectively.

When we classified all geographical populations into two subspecies, the genetic distance between *O. hupensis hupensis* from five provinces along the middle and lower reaches of Yangtze River and *O. hupensis robertsoni* from mountainous regions of Sichuan and Yunnan provinces was apparent (*F*st = 0.810, *P*<0.001) and the gene flow was limited (Nm = 0.117, *P*<0.001), indicating that the diversity between the two subspecies is significantly obvious.

In neutrality analyses, strong selection has been observed in *O. hupensis robertsoni* either with Tajama's *D* or Fu's *F* test (*P*>0.1). Although limited deviation has been observed for *O. hupensis hupensis* (*Fs* = −12.51, *P* = 0.011). Except a real departure from neutrality, the same pattern can be obtained after a recent population expansion when equilibrium between gene flow and drift has not yet to be reached [31],[32].

### Mismatch distribution

**Figure 2:**
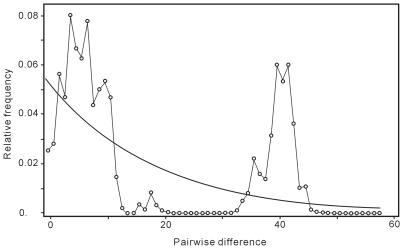
Mismatch distribution of the number of difference between all possible pairs of haplotypes. Comparison between the empirical distribution (◯—◯) and the expected distribution (—) under expansion model.

Through mismatch distribution analysis, the observed (empirical) distribution of haplotype pairwise differences followed a multimodal, ragged pattern, deviating significantly from the expected curve reflecting population expansion (*P* = 0.002) (Fig. 2). This pattern suggests that *O. hupensis* has already differentiated genetically in mainland China, which in turn verified the diversity between *O. hupensis hupensis* and *O. hupensis robertsoni*. In contrast, *O. hupensis hupensis* displayed a smooth unimodal mismatch distribution, which is consistent with the expected values of an expanding population, supporting the latter possibility in the neutral analyses for *O. hupensis hupensis*, that is, *O. hupensis hupensis* has a recent population expansion while equilibrium between gene flow and drift has not yet to be reached.

### Phylogenetic relationship

**Figure 3:**
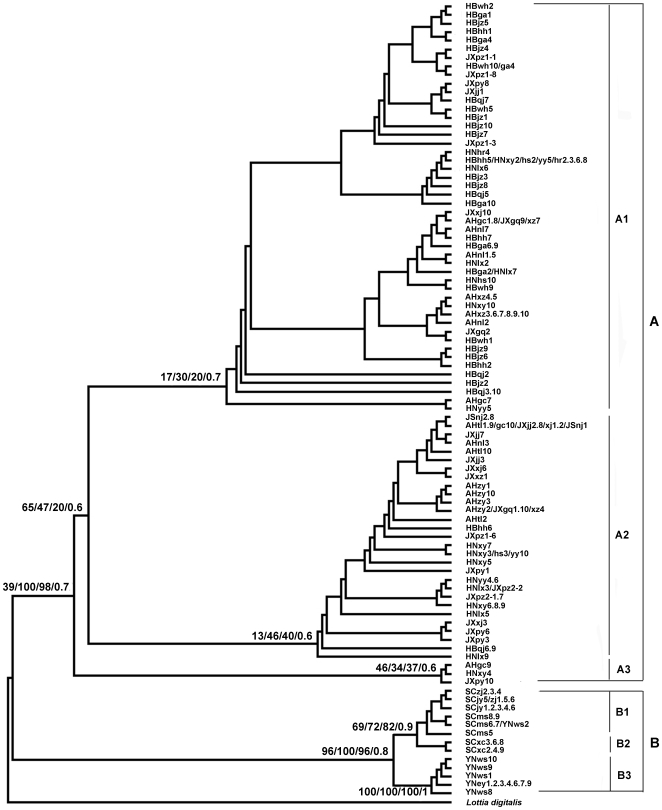
Phylogram of *Oncomelania hupensis* based on ITS1–ITS2 sequences. Values beside the branch represent proportions of 1000 bootstrap pesudoreplicates and posterior probabilities in which the node was recovered for ML/NJ/MP/BI, respectively. The letters after each branch represent different sampled individuals coded by locality letter followed by sample isolate number, and samples in one branch shared one haplotype.

Tree topologies generated by different building methods using NJ, ML, MP and BI were similar. Two distinct clades (clades A and B) were supported by high posterior probability or bootstrap values at key nodes (Fig. 3, ML tree). Clade A includes all haplotypes from five provinces including Anhui, Hubei, Hunan, Jiangxi and Jiangsu along the middle and lower reaches of the Yangtze River, and within this clade, a deep divergence was observed and it is quite obvious that three subclades, shown as A1, A2 and A3 can be recognized; but there is no distinct geographical relationship or phenotype characters, and posterior probabilities were low amongst the subclades. Clade B contains only haplotypes from mountainous regions in Sichuan and Yunnan provinces, and two subclades (subclade B1+B2 and subclade B3) were formed and supported by high posterior probabilities, which represent haplotypes from Sichuan and Yunnan provinces, respectively, except one shared haplotype from SCms and YNws populations in Sichuan and Yunnan provinces, respectively.

### Haplotype network and branch time

**Figure 4:**
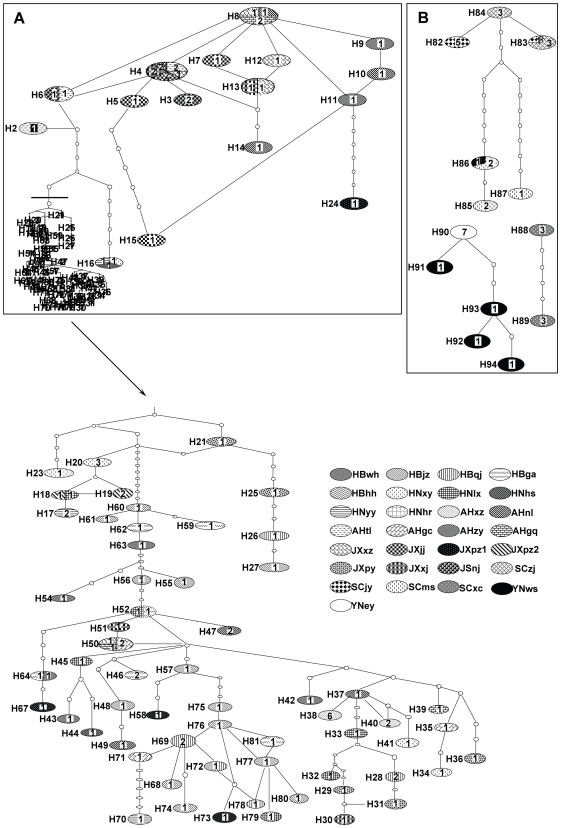
Unrooted parsimony network of ITS–ITS2 sequence haplotypes for *Oncomelania hupensis*. Ovals indicate sampled haplotypes, which have designated numbers beside them. Numbers inside ovals indicate sample numbers from different geographical populations which shared the same haplotype. Small circles indicate unsampled or extinct haplotypes. Each connection represents one mutational step. A and B (B1, B2 and B3) refer to the corresponding clades in Fig. 3, respectively. The branch under the dark line in clade A is expanded below, as indicated by the arrow.

The haplotype network constructed by statistical parsimony had similarity at least to some extent to the phylogenetic tree, especially in that the haplotype networks between samples from lake/marshland and hill regions in five provinces along the middle and lower reaches of Yangtze River and those from mountainous regions of Sichuan and Yunnan provinces were so diversified (Fig. 4). But, haplotypes from the middle and lower reaches of Yangtze River were mixed into a reticulate topology of evolution, forming into cluster A, which was reflected as clade A in the phylogenetic tree (Fig. 3). It was, however, impossible to further group these haplotypes. For haplotypes from Sichuan and Yunnan provinces, three separate clusters were detected (Fig. 4), which are completely consistently with the subclades B1, B2 and B3 in the phylogenetic tree (Fig. 3).

Based on the substitution rates for invertebrate ITS sequences ranging from 0.4% to 1.2%/Myr [33]–[35], it is estimated that the divergence between *O. hupensis hupensis* and *O. hupensis robertsoni* is about from 2±0.29 to 6±0.15Ma (D_xy_ = 0.048±0.0070).

## Discussion

This study demonstrated distinct genetic differentiation of *O. hupensis* from 29 geographical populations collected from 7 provinces in mainland China, accounting for most ecological habitat types for *O. hupensis* in endemic areas of China. Phylogenetic analyses revealed two distinct well-supported clades: One included all samples from lake/marshland and hill regions in five provinces along middle and lower reaches of the Yangtze River, the other one included samples from mountainous regions of Sichuan and Yunnan provinces. The average genetic divergence between the two clades is up to 0.81 based on *F*st, which is considered to be ‘very great’ by following the views of Wright [36]. Furthermore, the haplotype network revealed no connection between *O. hupensis hupensis* populations from lake/marshland and hill regions and *O. hupensis robertsoni* populations from mountainous regions, which also confirmed the genetic diversity of *O. hupensis* in mainland China geographically. The significant genetic differentiation was also reflected in the multimodal distribution in the mismatch analysis.

The genetic diversity of *Oncomelania* in China was previously examined by using COI [3],[37], Cytb [15], 16S rDNA [37] sequences and other methods such as AFLP [12],[13], and it has been shown that *O. hupensis hupensis* and *O. hupensis robertsoni* are genetically different. As revealed in the phylogenetic tree and haplotype network in the present study, *O. hupensis robertsoni* from Yunnan province differed genetically from those in Sichuan province, despite a shared haplotype from YNws and SCms which may need some further research. Li et al. [14], also using ITS sequences, found that *O. hupensis robertsoni* from Sichuan and Yunnan provinces were clustered into separate clades, although they were included in a larger clade, as observed in the present study. ITS, flanking sequences emanated from non-coding rDNA region, has a relatively fast evolutionary rate, and can be employed for investigating genetic differentiation and phylogeny of closely related species [38],[39]. In consideration of the ITS potential for heterozygote analysis [40], the large amount of samples used in the present study may stabilise the estimation of genetic variation and give more statistical confidence in the results [12],[41].

In other studies (data not shown here), we found that the complete mitochondrial DNA sequences had 10.3% genetic distance between *O. hupensis hupensis* and *O. hupensis robertsoni*, which may also reveal high genetic diversity between these subspecies. This information, to some extent, confirms the existence of wide genetic diversity for *O. hupensis* in mainland China. Although direct molecular evidence has not been previously available for the genetic diversity of *O. hupensis*, several authors [1]–[4],[11],[14] have considered that *O. hupensis* in mainland China can be separated into several subspecies, for example, *O. hupensis hupensis* from middle and lower reaches of Yangtze River, and *O. hupensis robertsoni* from mountainous regions of Sichuan and Yunnan provinces. It can then be concluded that these two subspecies differ not just in phenotypes and ecological habitats, but also genetically. Cross et al. [42] and He et al. [43] even showed that *O. hupensis* from different regions differed in their susceptibility to the same strain of *Schistosoma japonicum*, which may also have been reflected in genetic diversity of the snail intermediate hosts.

Ecological habitat and geographical distance were found to have some impact on genetic diversity of *O. hupensis* in mainland China [e.g. 14]. It has been suggested that *O. hupensis* evolved during its dispersal down the Yangtze River system, which would lead to genetic distance increasing with geographical distance [3]. Zhou et al. [12],[13] also found significant spatial genetic structure among 25 snail populations from 10 provinces in mainland China using AFLP, which was also verified by Li et al. [14] using ITS and 16S markers with a total of 30 individuals investigated in 13 localities. The habitats of *O. hupensis* in the middle and lower reaches of the Yangtze River include lake/marshland regions and hill regions, both of which have extensive physical connections with the Yangtze River through channels or in low floodplains beside the Yangtze River. With frequent floodings of the Yangtze River, snails in these habitats can be dispersed and subsequently deposited widely in various localities. The accumulation of mixed sources of snails can then generate genetically diversified populations of snails, leading to the existence of various haplotypes as observed in the present study. As found by Wilke et al. [3], ribbed-shelled snails and smooth-shelled snails but with varix on shell in the middle and lower reaches of Yangtze River were also clustered together in the phylogenetic tree. Whether this is the effect of potential heterozyges for ITS or not needs to be further investigated. The three subclades within the clade containing all samples, including those smooth-shelled snails with varix obtained in the middle and lower reaches of the Yangtze River may also indicate the genetic diversity of *O. hupensis hupensis*; it is therefore necessary to further investigate the genetic diversity of these snails by using more powerful tools and by covering more areas in the region.

In Sichuan and Yunnan provinces in the upper reaches of the Yangtze River, *O. hupensis robertsoni* are distributed in mountainous areas, and are not subjected to flood influence as much as in the middle and lower reaches of the river [44]. It is interesting to see that a relatively lower number of haplotypes were found in this region as compared with *O. hupensis hupensis*. Overall, these mountainous populations were genetically different from the populations in the middle and lower reaches of the river, as shown by phylogenetic trees, haplotype networks and genetic distance analyses. It thus appears likely that there has been certain degree of isolation for these mountainous populations. Wilke et al. [37] also found the diversity trend of *O. hupensis robertsoni* by COI and 16S rRNA sequences. It may also be possible that continuous control efforts, such as routine molluscicides in China, which have been used to control snails for about fifty years, might have imposed some effect on population genetics of these snails [45]. The diversity found in populations from Sichuan and Yunnan provinces may also need to be further clarified by obtaining more samples and by using more powerful molecular markers such as microsatellites.

About the origin and evolution history of *Oncomelania*, Davis [46] proposed a Gondwanan origin for the Pomatiopsidae, with rafting to mainland Asia via the Indian Craton after break-up of Gondwanan and colonization of South-East Asia and China. It is hypothesized [16],[47] that *Oncomelania* snails, arrived in southwestern China from Indian before the second (major) Tibetan orogeny (2.5 Ma), then evolved and spread down their respective river systems, to mainland of China, Indonesia and Philippines. Although mutation rate calibrations using fossil data is impossible here, many studies have demonstrated the confidence that molecular data can provide reasonable estimates of divergence time. Our data suggested that the two subspecies began to diverse as early about 2–6 Ma based on the invertebrate ITS substitution rate range. We did not find any strong molecular and fossil evidences about *Oncomelania* evolution, but the reported *Oncomelania* fossil found in Guangxi (1 Ma) by Odhner in 1930 and geological movement make this diversification time reasonable. It provides a new insight into the *Oncomelania* evolution history although the substitution rate needs to be verified with new fossil and molecular data in future study.

Davis et al. [48] speculated that, as *Oncomelania* snail populations form have diverged genetically, so must their associated schistosomes or else become regionally extinct. East Asian schistosomes and snails in the Pomatiopsidae have been considered as the only example of schistosome-intermediate host snail coevolutionary model [49], and a recent study also revealed that *S. japonicum* in mainland China can be highly genetically diverse, especially between populations from the lake/marshland lowland localities and populations from highland mountainous localities [50]. The continuous dispersal of the snails, probably as well as their schistosome parasites, in the middle and lower reaches of the Yangtze River may have considerable epidemiological, medical and evolutionary implications for the schistosome-snail system and schistosomiasis, as also suggested by Ross et al. [9]. It would be interesting, and necessary, to understand the population genetic diversity of the parasites and their intermediate hosts in greater detail throughout their distributions.

In summary, by cloning ITS1–ITS2 sequences, it has been shown that *O. hupensis* is highly genetically diverse. This clear and distinct genetic diversity in snail intermediate hosts may have strong implications in genetic diversity of schistosomes in China, and further studies on comparative phylogeography of the host-parasite system and also on their population genetics are necessary to understand the complexity of host-parasite population structures and evolutionary, if not co-evolutionary, relationships.

## Supporting Information

Alternative Language Abstract S1 Chinese translation of the abstract by PN.
(0.06 MB PDF)
