# Supplementary material for: Distinct Genetic Diversity of Oncomelania hupensis, Intermediate Host of Schistosoma japonicum in Mainland China as Revealed by ITS Sequences
Source: PLoS Negl Trop Dis. 2010 Mar 2;4(3):e611. doi: 10.1371/journal.pntd.0000611 (PMC2830461; doi:10.1371/journal.pntd.0000611)
Supplement: Alternative Language Abstract S1 — Chinese translation of the abstract by PN. (0.06 MB PDF) [file pntd.0000611.s001.pdf]

摘要

湖北钉螺（*Oncomelania hupensis*）是日本血吸虫的中间寄主，通常可以分为两种表型，一种是肋壳，一种是光壳。在中国分布的湖北钉螺主要是肋壳钉螺，而且这种表型的钉螺只在中国分布。此外，光壳钉螺在中国也广泛分布。本研究中，我们利用核糖体 ITS1 和 ITS2 序列，对来自中国大陆长江中下游地区的肋壳钉螺和光壳（但壳上有螺层（varix）的）钉螺，以及来自云南、四川的光壳钉螺进行遗传变异分析，发现长江中下游的钉螺与云南和四川的钉螺有明显的遗传差异，它们之间没有共有的单倍型。此外，云南和四川的光壳钉螺在系统发育树上聚成不同的分枝，长江中下游的钉螺则聚为三枝，表明湖北钉螺在中国有较高的遗传差异，它们是否与日本血吸虫有协同进化关系有待进一步研究。
